# A retrospective examination of mean relative telomere length in the Tasmanian Familial Hematological Malignancies Study

**DOI:** 10.3892/or.2014.3568

**Published:** 2014-10-24

**Authors:** NICHOLAS B. BLACKBURN, JAC C. CHARLESWORTH, JAMES R. MARTHICK, ELIZABETH M. TEGG, KATHERINE A. MARSDEN, VELANDAI SRIKANTH, JOHN BLANGERO, RAY M. LOWENTHAL, SIMON J. FOOTE, JOANNE L. DICKINSON

**Affiliations:** 1 Menzies Research Institute Tasmania, University of Tasmania, Hobart, TAS 7000, Australia; 2 Department of Genetics, Texas Biomedical Research Institute, San Antonio, TX 78245-0549, USA; 3 Royal Hobart Hospital, Hobart, TAS 7001, Australia; 4 School of Medicine, University of Tasmania, Hobart, TAS 7000, Australia; 5 Department of Medicine, Monash Medical Centre, Faculty of Medicine, Nursing and Health Sciences, Monash University, Clayton, VIC 3168, Australia; 6 John Curtain School of Medical Research, Australian National University, ACT 2601, Australia

**Keywords:** telomere length, hematological malignancies, familial cancer

## Abstract

Telomere length has a biological link to cancer, with excessive telomere shortening leading to genetic instability and resultant malignant transformation. Telomere length is heritable and genetic variants determining telomere length have been identified. Telomere biology has been implicated in the development of hematological malignancies (HMs), therefore, closer examination of telomere length in HMs may provide further insight into genetic etiology of disease development and support for telomere length as a prognostic factor in HMs. We retrospectively examined mean relative telomere length in the Tasmanian Familial Hematological Malignancies Study using a quantitative PCR method on genomic DNA from peripheral blood samples. Fifty-five familial HM cases, 191 unaffected relatives of familial HM cases and 75 non-familial HM cases were compared with 758 population controls. Variance components modeling was employed to identify factors influencing variation in telomere length. Overall, HM cases had shorter mean relative telomere length (P=2.9×10^−6^) and this was observed across both familial and non-familial HM cases (P=2.2×10^−4^ and 2.2×10^−5^, respectively) as well as additional subgroupings of HM cases according to broad subtypes. Mean relative telomere length was also significantly heritable (62.6%; P=4.7×10^−5^) in the HM families in the present study. We present new evidence of significantly shorter mean relative telomere length in both familial and non-familial HM cases from the same population adding further support to the potential use of telomere length as a prognostic factor in HMs. Whether telomere shortening is the cause of or the result of HMs is yet to be determined, but as telomere length was found to be highly heritable in our HM families this suggests that genetics driving the variation in telomere length is related to HM disease risk.

## Introduction

Telomeres are DNA-protein structures at chromosome ends consisting of repeating hexameric nucleotide sequences of TTAGGG (1). The primary role of the telomere is to cap chromosome ends to prevent aberrant recombination as a result of exposed chromosomal DNA; making telomeres essential for maintenance of genomic integrity (2). With each cell division telomeres shorten due to the incomplete DNA replication of chromosome ends by DNA polymerases eventually triggering cell senescence or apoptosis to prevent further shortening and exposure of chromosomal DNA (3). The telomerase complex can counteract telomere shortening in actively dividing cells by catalyzing the addition of TTAGGG repeats to chromosome ends (3,4), however, this is not a full restoration and telomeres progressively shorten with age (3,5,6). A number of studies have reported an association between telomere length in lymphocytes and an increased risk of age-related diseases including cancer (7,8). To date, the main understanding of the role of telomeres in cancer is that excessive telomere shortening leads to increased genetic instability and chromosomal end-to-end fusions (2,9), which then leads to a malignant cell transformation (9).

Studies of monozygotic and dizygotic twins and large families have established a genetic component to the determination of telomere length. Estimates of the heritability of telomere length ranges between 78 and 82% in studies of twins and sibling pairs (which generally produce inflated heritability estimates) (10,11) and 44% in a study of large Amish families (12). Although it has been proposed that the heritability of telomere length can be accounted for by shared environmental factors (13) the consensus is that telomere length is primarily determined by parental inheritance including at least partial inheritance of chromosome-specific telomere lengths (14,15). This view is strongly supported by mouse models of telomere length inheritance (16).

Telomere length is a proposed risk factor for cancer given its importance in maintaining genomic integrity (2,9) and shorter telomeres have been shown to be associated with a range of cancers (7,8,17). The proposed role of telomere length as a cancer risk factor, coupled with its high heritability, raises the idea that telomere length may also be important in familial cancers such as familial hematological malignancies (HMs). Additionally, inherited changes in telomere length as a result of mutations in the telomere maintenance genes *TERT* and *TERC* have been identified in four HM families with myelodysplastic syndrome and acute myeloid leukemia (MDS-AML) (18). Therefore, telomere length may be a risk factor for other HM subtypes.

Hematological malignancies arise as a result of the neoplastic transformation of cells involved in hematopoiesis and include a broad range of subtypes of leukemias, lymphomas and myelomas (19). A well-established risk factor for HMs is family history indicating that familial HMs have an underlying genetic component. For example, a population-based study of the Swedish Cancer Registry showed an 8.5-fold increase in the risk of developing chronic lymphocytic leukemia (CLL) in first-degree relatives of CLL patients as well as an increased risk of developing other HM subtypes providing evidence for a shared genetic etiology across HM subtypes (20).

There is accumulating evidence that implicates telomere length as an important factor in the development of a range of HMs. This includes studies of several different HM subtypes where shorter telomeres were found in circulating tumor cells (21–26). While these studies have revealed important insights into telomere dynamics in circulating tumor cells there has been less focus on prospective and retrospective studies of pre-disease and remission telomere lengths respectively in HM patients. Indeed, one prospective study of telomere length in pre-disease blood samples surprisingly showed that longer telomere length was associated with a future risk of NHL (non-Hodgkin lymphoma) (27). Furthermore, in a study of chronic leukemia, Mansouri and colleagues (28) elegantly showed that telomere length has potential as a clinical prognostic marker in HMs. In their study, patients with shorter telomeres were associated with high-risk genetic markers and in patients with otherwise good prognostic markers, telomere length was an independent prognostic factor that subdivided the good prognosis group into groups with distinct outcomes. Therefore, there is potential for telomere length to be a clinically relevant prognostic risk factor for HMs.

The aim of the present study was to explore whether telomere length is involved in familial HMs and to find new evidence supporting telomere length as a prognostic risk factor for HMs. To this end, we examined telomere length in the Tasmanian Familial Hematological Malignancies Study (TFHMS); a genetic study comprising large Tasmanian families with multiple cases of HMs and a collection of population matched non-familial HM cases (29–31) and controls (32,33). Previously, similar collections have focused on families with one predominant subtype of HM. The TFHMS includes families with a dense aggregation of several HM subtypes across multiple generations; for example family LK2042 includes 32 cases in five generations (Table I).

The strength of a familial approach to examining telomere length lies in the enrichment of the shared genetic backgrounds between related individuals as related cases are likely to share genetic variants contributing to variation in telomere length which may in turn be affecting their risk of developing HMs. In the present study, we used the TFHMS to measure the heritability of telomere length as a quantitative trait in the study families and then examine whether HMs account for measured variation in telomere length.

## Materials and methods

### Ethics statement

The TFHM study was approved by the Human Research Ethics Committee (Tasmanian network), reference number: H8551, and written informed consent was obtained from all participating individuals.

### The Tasmanian Familial Hematological Malignancies Study

As previously described (29) during the period 1972–1980 all patients with HMs diagnosed in Tasmania (the island state of Australia) were invited to participate in a population-based study examining the association of occupation and place of residence with risk of development of myeloproliferative and lymphoproliferative disorders (34,35). Using a genealogical database at the Menzies Research Institute Tasmania the individuals participating in the original population-based study were linked to both current generations and records from the Tasmanian Cancer Registry, which has documented cases of HMs since 1978. Family members provided further information through questionnaires and personal interviews. This allowed us to form pedigrees of Tasmanian families with multiple cases of HMs as well as a collection of HM cases with no reported family history of disease (Table I).

Confirmation of diagnosis was, where possible, obtained for all cases and in particular 13 study families were classified by a single experienced hematologist (E.M.T.) according to the 2008 World Health Organization classification (19) as previously described (29). For the remaining study families, case diagnosis was obtained from the Tasmanian Cancer Registry records and by review of available pathology reports of cases that consented to participate in the TFHMS. More extensive clinical information is not currently available due to the multi-center and multi-specialist nature of the original data collection.

### Study samples

In this TFHM-based study we used DNA obtained from peripheral blood samples from 55 familial HM cases, 191 unaffected relatives of familial cases and 75 non-familial cases. DNA from 40 TFHMS families was available for the present study with samples available from both HM cases and unaffected relatives in 14 families. The remaining families were comprised of samples from HM cases with a known family history of disease alone or from unaffected relatives of HM cases. Of the 191 unaffected relatives, 171 were first-degree relatives of HM cases and the remaining subjects were more distantly related or spouses. For HM cases, DNA was collected from 1 month to 64.9 years post HM diagnosis (mean, 9.9 years). Population controls were recruited randomly from the Tasmanian electoral role (n=758) through the TASCOG study (33) (a population-based study of gait in older Tasmanians) or obtained from the control samples in a Tasmanian familial prostate cancer case-control study (32) both conducted at the Menzies Research Institute Tasmania. Details concerning the participants in this study are shown in Table II, and the distribution of HM case subtypes in this study is summarized in Table III. Non-familial HM cases had no self-reported family history of HMs and did not appear in any of our study families after thorough genealogical examination. Frequent updates from the Tasmanian Cancer Registry were used to monitor the occurrence of HMs in the unaffected relatives that are part of this study. Population control DNA samples were also extracted from peripheral blood samples in the same laboratory, using the same methodology as the TFHMS samples. Genomic DNA was extracted from peripheral blood samples using the Nucleon BACC 3 DNA Extraction kit (GE Healthcare).

### Telomere length measurement

We investigated the mean relative telomere length in peripheral blood samples using a slightly amended protocol for a validated monochrome multiplex quantitative PCR method outlined by Cawthon (36). This method measures the relative telomere length by calculating the ratio, T/S, between telomere repeat copy number amplification (T) and the amplification of a single-copy gene, albumin (S). The average T/S ratio was obtained as the mean of the triplicate measurements for each sample. Individual measurements were excluded from the average T/S ratio calculation when the replicate failed or a large standard error was observed. The coefficient of variation calculated across all assay plates using repeated cross-plate samples was 3.4%.

Telomere length measurement was performed in 10 μl volumes using a LightCycler 480 in a 96-well plate format. Each 96-well plate contained a six point standard curve 2, 5, 15, 50, 100 and 150 ng, a unique sample common to each plate, a no template control and 24 unknown case/control samples all repeated in triplicate, with 1.6% sample replication across plates. The genomic DNA used for the standard curve was from a 27-year-old female control study participant.

Final reagent concentrations were 5 ng of genomic DNA, primer telg 200 nM (5′-ACACTAAGGTTTGGGTTTGGGT TTGGGTTTGGGTTAGTGT-3′), primer telc 700 nM (5′-TG TTAGGTATCCCTATCCCTATCCCTATCCCTATCCCTAA CA-3′), primer albu 500 nM (5′-CGGCGGCGGGCGGCG CGGGCTGGGCGGAAATGCTGCACAGAATCCTTG-3′), primer albd 500 nM (5′-GCCCGGCCCGCCGCGCCCG TCCCGCCGGAAAAGCATGGTCGCCTGTT-3′), AmpliTaq Gold (Applied Biosystems) 0.625 U, GeneAmp 10× PCR buffer (Life Technologies) containing 50 mM KCl, 10 mM Tris-HCl pH 8.3 and 1.5 mM MgCl_2_, 1 mM DTT, 1 M Betaine (Sigma-Aldrich), 0.0025 mM Syto9 (Life Technologies) and 0.25 mM of each dNTP (Bioline). Cycling conditions were as follows: 95°C for 15 min, 2 cycles of 94°C for 15 sec, 49°C for 60 sec; four cycles of 84°C for 20 sec, 59°C for 30 sec, then 40 cycles of 94°C for 15 sec, 59°C for 30 sec with signal acquisition for telomere repeat copy number amplification, 84°C for 30 sec, then 85°C for 20 sec with signal acquisition for albumin amplification. A melting curve was generated for each plate. Ct values were calculated using LinRegPCR (37) and a standard curve was generated for both the telomere and albumin PCRs. A linear regression of the standard curve measurement values was used to correct for any variation in fluorescence levels derived from small fluctuations in DNA concentration. The equations from the linear regression of each standard curve were then used to calculate the log(DNA) value for the unknown case/control samples.

### Statistical analysis

Average T/S ratios greater than 4 standard deviations from the control mean were excluded as outliers. Mean relative T/S ratios with 95% confidence intervals (CI) are reported in Table II. For analysis mean relative T/S ratios were transformed to fit a normal distribution using the inverse-normalization option in SOLAR (version 6.6.2) (38,39) to prevent non-normal distribution errors. In order to fully utilize the extended pedigree study design, correct for relatedness, and to maximize the information provided by telomere length as a quantitative trait we used variance components modeling in SOLAR (38,39) to determine the heritability of telomere length (adjusting for kinship and significant covariates) and to calculate the association between telomere length and disease. The primary benefit to using SOLAR is its ability to incorporate relatedness through the use of a kinship matrix and to fully utilize the quantitative trait data, which increases the power and accuracy of the trait heritability calculation.

**Figure 1:**
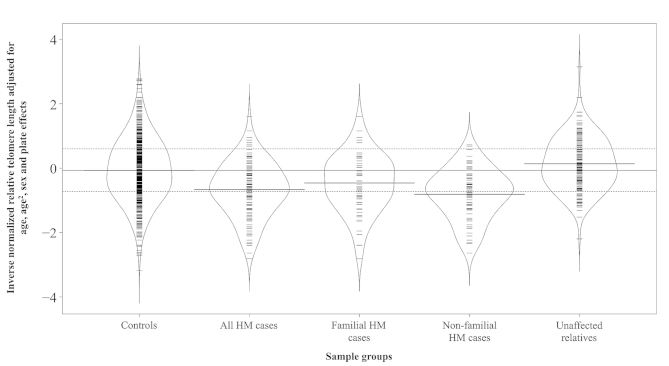
Bean plot quartile analysis of adjusted inverse normalized relative telomere lengths. The adjusted inverse normalized relative telomere length for each group is displayed as a bean plot with individual sample measurements as lines within the bean and the overall distribution of all samples in each group shown. Horizontal bars for each bean indicate the mean of each group. The solid line and dashed lines show the mean and interquartile range of the control group.

Sex, age, age^2^ and their interactions were included as covariates in all relevant analyses. Potential batch effects were adjusted for by applying household modeling (38,39) by coding each assay plate as a separate household. SOLAR has been previously used in the analysis of telomere length in related individuals (11,12,40). The algorithms utilized in SOLAR for the analysis of quantitative traits in related individuals are more appropriate to employ in the present study than a more traditional approach of analyzing quantitative traits using percentiles or quartiles. Nevertheless, we also present observations from a quartile analysis of inverse normalized relative T/S ratios adjusted for age, sex and batch effects in SOLAR with quartiles defined from the adjusted T/S ratios in the control population (Fig. 1). Bean plots in Fig. 1 were constructed using the R package ‘beanplot’ (41).

## Results

Mean relative telomere length in familial and non-familial HM cases, unaffected relatives and control subjects was measured by monochrome multiplex quantitative PCR. Using SOLAR we found that the heritability of mean relative telomere length was 62.5% (P=4.7×10^−5^, SE=0.14). The removal of HM cases (n=130) from analysis only marginally altered the heritability of mean relative telomere length (75.5%; P=1.2×10^−5^, SE=0.15).

The use of variance components modeling in SOLAR permits appropriate statistical analyses inclusive of familial relationships. These analyses revealed that disease status was significantly associated with mean relative telomere length (Table IV, primary analysis model 1; P=2.9×10^−6^) with HM cases having shorter mean relative telomere length when compared with unaffected individuals. We conducted a separate analysis distinguishing familial and non-familial cases. Familial cases and non-familial cases each had significantly shorter mean relative telomere length (Table IV; primary analysis model 2, P=2.2×10^−4^ and P=2.2×10-5, respectively).

The most frequent type of HM diagnosed in the present study was mature B cell neoplasms (MBCNs; Table III). Analysis of MBCNs as one group and HMs other than MBCNs as a second group (combined due to the small numbers of other subtypes) showed that both groupings had shorter mean relative telomere length than unaffected individuals from both study families and population controls (Table IV; primary analysis model 3, P=3.5×10^−5^ and P=9.3×10^−5^, respectively). These groupings were then divided according to whether the HM case was familial or non-familial. Analysis showed that all HM case subgroupings maintained significantly shorter mean relative telomere length (Table IV; primary analysis model 4). An analysis using specific HM subtypes was not possible due to not having enough statistical power at this level of HM classification with small numbers of HM subtypes (Table III).

Variance components modeling also identified age and sex (Table IV; primary analysis model 1, P=4.8×10^−8^ and P=4.0×10^−3^, respectively) as significant covariates for mean relative telomere length variation across all models. Mean relative telomere length declined with age and males had shorter telomeres than females. Age^2^ was also a significant covariate, indicating that the decline in mean relative telomere length with age has a non-linear component (Table III; primary analysis model 1, P=8.0×10^−3^).

Four sub-analyses of the primary data were also performed to determine whether particular features of the study population were contributing to the disease associations found in the primary analysis models (Table IV). Sub-analyses included exclusion of HM cases, controls and unaffected relatives 80 years or older (n=126), exclusion of CLL cases (n=24), exclusion of cases with samples collected within two years of diagnosis (n=24) as well as all three exclusions together (n=162, some individuals were in multiple exclusion categories). In each sub-analysis the principle findings from the primary analysis models were maintained.

Categorization of cases into quartiles of mean relative telomere length determined from the distribution of age, sex and batch effect adjusted mean relative telomere length in controls (Fig. 1) showed that 43.1% of HM cases were in the lowest quartile of mean relative telomere length (below the lower interquartile dashed line), with 36.4% of familial HM cases and 48% of non-familial HM cases in the lowest quartile, whilst only 13.1% of unaffected relatives were in the lowest quartile. Similarly a low percentage of cases (5.4%) were in the longest quartile of mean relative telomere length (above the upper interquartile dashed line) whereas 28.3% of unaffected relatives were in the longest quartile. A clear trend for shorter mean relative telomere length in a higher percentage of HM cases was observed but this analysis did not permit familial relationships to be included in the analysis.

## Discussion

These analyses determined that mean relative telomere length is highly heritable within the TFHMS families supporting previously reported heritability estimates in non-disease families (10–12). Our finding that mean relative telomere length was shorter in both familial and non-familial HM cases indicates that telomere length is likely to be important in the genetic etiology of HMs. A previous study of mean relative telomere length in familial myelodysplastic syndrome MDS-AML has shown that affected individuals from four small families had shorter telomeres concurrent with mutations in the telomerase gene *TERT* and its RNA component *TERC* (18). Of the five cases across the four families reported to have shorter telomeres, two had aplastic anemia, two had MDS and one had MDS-AML. The present study extends the findings of Kirwan and colleagues (18) to that of large families with multiple HM subtypes finding new evidence of the involvement of telomere length in both familial and non-familial HMs.

Age, sex and age^2^ as covariates explained a proportion of the variation in mean relative telomere length in the present study. This is in keeping with telomere length declining with age and males having shorter telomeres than females (7). A significant age^2^ indicates a non-linear component in the age-related telomere length decline, a finding in line with a recent report showing a differential rate of decrease in telomere length over different age ranges (42). Our population controls did have a higher percentage of males, which could be suggested to be driving the association with sex, however, SOLAR was used to correct the mean relative telomere length for sex effects.

An important caveat with our retrospective study is that the finding of shorter mean relative telomere lengths in HM cases could also be related to disease susceptibility, treatment or the disease process. The present study did not have the necessary clinical information to appropriately analyze these factors. Currently, the literature surrounding the role of chemotherapeutic agents in telomere shortening remains controversial and inconclusive. Several studies in both HMs and other cancers such as breast cancer have shown that telomere length is unaffected when comparing pre- and post-chemotherapy measurements, when comparing patients who receive chemotherapy to those that do not or when comparing telomere length between patients and population controls (25,28,43,44). Other studies show a heterogeneous effect of chemotherapy on telomere length (45–47). It could be concluded from these reports and others that chemotherapy has no consistent influence on telomere length in blood cells particularly when examining multiple chemotherapeutic treatment regimens.

A second consideration is that shorter telomeres in HM cases could be the result of malignant cell DNA within the genomic DNA sample. We recognize that circulating malignant cells can be present for many years in chronic HM subtypes such as CLL. Based on the clinical diagnoses of HM cases in our study, we conducted two additional sub-analyses of the primary data. In the one analysis we removed all CLL cases (n=24) on the basis that DNA obtained from blood of cases with this subtype of HM was likely to contain DNA from diseased cells (Table IV). In the second analysis we removed all cases for which blood samples were obtained for DNA within 2 years of diagnosis (n=24; Table IV). Repeating the variance components modeling in these two analyses maintained the key significant associations with HM disease, suggesting that circulating disease did not contribute to the telomere length associations we have identified. In an additional sub-analysis we excluded all HM cases, controls and unaffected relatives (n=126) aged 80 years and above on the basis that the population HM risk increases with age. This did not change the principle findings of shorter telomeres in familial and non-familial HM cases nor did a final combined sub-analysis excluding individuals from all 3 sub-analyses. All cases, controls and unaffected relatives were included in the primary analysis models reported in Table IV.

In conclusion, our analyses showed for the first time that mean relative telomere length is heritable in large HM families with multiple generations affected by multiple subtypes of HMs, indicating a strong genetic effect driving trait variation. We also showed that both familial and non-familial HM cases from the same population had shorter mean relative telomere length. Taken together, the results from this retrospective study provide new evidence that mean relative telomere length is an important genetic factor in a wide range of HM subtypes and in individuals with and without a family history of disease. These findings contribute further support to the use of telomere length as a prognostic risk factor for HMs

## Figures and Tables

**Table I:** Summary of the TFHMS families used in this study.

Family	Known HM cases	Generations with HM cases	HM cases with telomere length measurement	Unaffected relatives with telomere length measurement
LK0001	14	4	1	16
LK0002	15	3	1	5
LK0004	7	2	1	11
LK0016	18	5	2	19
LK0024	3	2	1	0
LK0026	6	2	1	5
LK0040	7	4	2	2
LK0051	21	5	3	26
LK0054	9	3	0	2
LK0065	8	2	0	8
LK0124	24	5	2	34
LK0132	5	2	0	7
LK0139	7	2	1	2
LK0153	9	2	3	2
LK0511	2	2	1	0
LK0512	2	1	1	0
LK0537	2	1	2	0
LK0546	2	2	1	0
LK0560	2	2	1	0
LK0561	2	2	1	0
LK0600	5	3	2	0
LK0625	4	2	2	0
LK0647	2	2	1	0
LK0672	3	3	1	0
LK0836	6	3	2	5
LK1155	2	1	1	3
LK2042	32	5	6	40
LK2447	3	2	1	2
LK6000	6	2	1	0
LK7739	2	1	1	0
LK7740	2	2	2	0
LK7743	3	2	2	0
LK7744	2	2	0	1
LK7748	2	2	1	0
LK7749	3	2	1	0
LK7750	4	2	2	0
LK7751	9	3	1	0
LK7754	3	1	1	0
LK7755	2	2	1	0
LK7768	2	1	1	0
Non-familial cases	-	-	75	1

HM, hematological malignancy; TFHM, Tasmanian Familial Hematological Malignancies Study.

**Table II:** Mean age, sex distribution and relative telomere length in the sample groups.

Sample group	N	Male sex, n (%)	Mean age (range)	Mean relative T/S ratio^a^ (95% CI)
Controls	758	578 (76.3)	67.51 (30.67–87.97)	0.64 (0.62–0.66)
Unaffected relatives of HM cases	191	77 (40.3)	61.65 (27.26–92.95)	0.73 (0.69–0.76)
All HM cases	130	73 (56.2)	65.14 (13.24–95.53)	0.53 (0.50–0.56)
Familial HM cases	55	32 (58.2)	64.45 (13.24–87.45)	0.57 (0.52–0.63)
Non-familial HM cases	75	41 (54.7)	68.79 (22.42–95.53)	0.50 (0.46–0.53)

a Mean relative T/S ratio is the ratio between telomere repeat copy number (T) and a single-copy gene, *ALB*, copy number (S), a measure of mean relative telomere length.

CI, confidence interval; HM, hematological malignancy. Mean age (range) is expressed in years.

**Table III:** Disease characteristics of study samples.

	HM familial cases, n (%)	HM non-familial cases, n (%)	All HM cases, n (%)
HM subtypes			
Acute lymphoblastic leukemia	2 (3.6)	0	2 (1.5)
Acute myeloid leukemia	5 (9.1)	8 (10.7)	13 (10.0)
Chronic myeloid leukemia	0	3 (4.0)	3 (2.3)
Essential thrombocythemia	1 (1.8)	1 (1.3)	2 (1.5)
Hodgkin lymphoma	5 (9.1)	4 (5.3)	9 (6.9)
Myelodysplastic syndrome	2 (3.6)	0	2 (1.5)
Myeloproliferative neoplasm	1 (1.8)	2 (2.7)	3 (2.3)
T-cell non-Hodgkin lymphoma	1 (1.8)	2 (2.7)	3 (2.3)
Mature B cell neoplasms			
Non-Hodgkin lymphoma unclassified	2 (3.6)	10 (13.3)	12 (9.2)
Chronic lymphocytic leukemia	12 (21.8)	12 (16.0)	24 (18.5)
Diffuse large B-cell lymphoma	4 (7.3)	10 (13.3)	14 (10.8)
Follicular lymphoma	4 (7.3)	9 (12.0)	13 (10.0)
Multiple myeloma	7 (12.7)	5 (6.7)	12 (9.2)
Other^a^	9 (16.4)	9 (12.0)	18 (13.8)
Total	55	75	130

a Other includes Burkitt lymphoma, hairy cell leukemia, lymphoma of mucosa-associated lymphoid tissue and Waldenström macroglobulinemia.

HM, hematological malignancy.

**Table IV:** Variance component modeling analysis of inverse normalized mean relative telomere length primary analysis and sub-analyses with exclusions.

Models and variables	Primary analysis P-values	≥80 years old excluded (n=126) P-values	CLL cases excluded (n=24) P-values	Possible malignant samples excluded^a^ (n=24) P-values	All exclusions applied (n=162) P-values
Model 1					
Age	4.8×10^−8^	7.5×10^−5^	1.6×10^−8^	6.9×10^−8^	3.4×10^−5^
Age^2^	8.0×10^−3^	0.04	6.0×10^−3^	0.01	0.07
Sex	4.0×10^−3^	0.01	7.0×10^−3^	2.0×10^−3^	0.01
All HM cases	2.9×10^−6^	7.3×10^−6^	2.9×10^−7^	1.1×10^−4^	4.6×10^−5^
% trait variance accounted for by model	10.07%	9.46%	10.38%	9.56%	9.38%
Model 2					
Age	4.3×10^−8^	5.1×10^−5^	1.6×10^−8^	7.4×10^−8^	3.7×10^−5^
Age^2^	8.0×10^−3^	0.04	6.0×10^−3^	0.01	0.07
Sex	3.0×10^−3^	0.01	6.0×10^−3^	2.0×10^−3^	9.0×10^−3^
Familial HM cases	2.2×10^−4^	1.0×10^−3^	1.6×10^−5^	0.01	8.0×10^−3^
Non-familial HM cases	2.2×10^−5^	6.9×10^−6^	7.1×10^−5^	3.3×10^−5^	2.7×10^−5^
% trait variance accounted for by model	10.62%	10.29%	10.55%	10.48%	10.00%
Model 3					
Age	4.7×10^−8^	7.2×10^−5^	1.7×10^−8^	7.7×10^−8^	3.7×10^−5^
Age^2^	8.0×10^−3^	0.04	6.0×10^−3^	0.01	0.07
Sex	4.0×10^−3^	0.01	6.0×10^−3^	2.0×10^−3^	0.01
MBCNs	3.5×10^−5^	7.8×10^−5^	5.5×10^−6^	5.7×10^−4^	3.8×10^−4^
HMs other than MBCNs	9.3×10^−5^	1.5×10^−4^	3.4×10^−5^	1.0×10^−3^	5.8×10^−4^
% trait variance accounted for by model	10.08%	9.50%	10.37%	9.56%	9.39%
Model 4					
Age	4.8×10^−8^	4.1×10^−5^	1.9×10^−8^	6.6×10^−8^	3.8×10^−5^
Age^2^	9.0×10^−3^	0.05	7.0×10^−3^	0.02	0.08
Sex	3.0×10^−3^	0.01	6.0×10^−3^	2.0×10^−3^	9.0×10^−3^
Familial MBCNs	0.02	0.04	3.0×10^−3^	0.18	0.07
Familial cases other than MBCNs	5.2×10^−4^	3.0×10^−3^	5.7×10^−4^	0.01	0.04
Non-familial MBCNs	2.4×10^−5^	1.5×10^−5^	1.3×10^−4^	4.8×10^−5^	1.5×10^−4^
Non-familial cases other than MBCNs	2.0×10^−3^	4.2×10^−4^	3.0×10^−3^	2.0×10^−3^	3.2×10^−4^
% trait variance accounted for by model	10.87%	10.58%	10.57%	10.45%	10.05%

P-values for the significance of each trait or covariate were derived from variance component polygenic modeling in SOLAR.

a HM case samples collected within ± 2 years of diagnosis were excluded.

CLL, chronic lymphocytic leukemia; HM, hematological malignancy; MBCNs, mature B-cell neoplasms.
